# Trajectories of oxygen saturation within 6–72 hours after birth in neonates at moderate altitude: a prospective longitudinal cohort study

**DOI:** 10.1007/s12519-023-00687-w

**Published:** 2023-02-13

**Authors:** Qu-Ming Zhao, Hong-Yan Chen, Shi-Xiu Li, Wei-Li Yan, Xiao-Jing Hu, Guo-Ying Huang

**Affiliations:** 1 Children’s Hospital of Fudan University, National Children’s Medical Center, 399 Wan Yuan Road, Shanghai, 201102 China; 2 Shanghai Key Laboratory of Birth Defects, 399 Wan Yuan Road, Shanghai, 201102 China; 3 Luchun County People’s Hospital, Luchun, China; 4 Research Unit of Early Intervention of Genetically Related Childhood Cardiovascular Diseases (2018RU002), Chinese Academy of Medical Sciences, Shanghai, China

**Keywords:** Critical congenital heart disease, Hypoxemic disease, Pulse oximetry screening, Pulse oxygen saturation

## Abstract

**Background:**

Trajectories of pulse oxygen saturation (SpO_2_) within the first few days after birth are important to inform the strategy for identifying asymptomatic hypoxemic disease but remain poorly substantiated at higher altitudes.

**Methods:**

We performed a longitudinal cohort study with consecutive neonates at a local hospital in Luchun County, China, at an altitude of 1650 m between January and July 2020. We repeatedly measured the pre- and post-ductal SpO_2_ values at 6, 12, 18, 24, 36, 48, and 72 hours after birth for neonates without oxygen supplements. All neonates underwent echocardiography and were followed up to 42 days after discharge. We included neonates without hypoxemic diseases to characterize the trajectories of SpO_2_ over time using a linear mixed model. We considered the 2.5th percentile as the reference value to define hypoxemic conditions.

**Results:**

A total of 1061 neonates were enrolled. Twenty-five had non-cardiac hypoxemic diseases, with 84% (21/25) presenting with abnormal SpO_2_ within 24 hours. One had tetralogy of Fallot identified by echocardiography. Among the 1035 asymptomatic neonates, SpO_2_ values declined from 6 hours after birth, reached a nadir at 48 hours, and tended to level off thereafter, with identical patterns for both pre- and post-ductal SpO_2_. The reference percentile was 92% for both pre- and post-ductal SpO_2_ and was time independent.

**Conclusions:**

A decline within 48 hours features SpO_2_ trajectories within the first 72 hours at moderate altitude. Our findings suggest that earlier screening may favorably achieve a benefit–risk balance in identifying asymptomatic hypoxemic diseases in this population.

## Introduction

Pulse oximetry has been used as a predischarge screening in newborns to identify hypoxemic diseases [1]. A comprehensive understanding of the dynamics of pulse oxygen saturation (SpO_2_) during the first few days after birth at altitudes could help inform the choice of the optimal timing and threshold values to identify hypoxemic diseases. Such knowledge is crucial for both critical congenital heart disease (CCHD) screening and appropriate oxygen therapy to avoid hyperoxia-related risk from excess use of supplemental oxygen [2, 3]. However, the data are scarce and are compounded by the different physiologic adaptations between newborns at higher altitudes and those at sea level [4, 5].

Niermeyer and colleagues**'** studies demonstrated that in healthy infants at high altitudes above 3100 m, mean SpO_2_ values fell within the first 48 hours and continued at least one week after birth [6–8]. A large cross-sectional study of 555 infants at a moderate altitude of 1800 m reported no changes in SpO_2_ within the first 24 hours**;** nevertheless**,** the highest value was within 6 hours [9]. These findings contrast with the presumably lower SpO_2_ values within 24 hours, raising concerns about whether the recommended time window of 24–48 hours for pulse oximetry screening for CCHD to avoid high false positives at sea level is appreciably applicable to higher altitudes [10, 11]. This issue is further complicated by the need for timely diagnosis of non-cardiac hypoxemia diseases; these diseases are likely to develop within the first 24 hours and can be severe and life threatening [10, 12, 13]. There are also uncertainties regarding the threshold values for CCHD screening or initiation of oxygen treatment [3]. Although lower threshold values of 90%–93% SpO_2_ have been used, they are selected experientially rather than derived from empirical data, which would be ideal [14–16]. Without knowing how precisely SpO_2_ changes over time, it is difficult to justify the optimal timing and low limits for identifying hypoxemic diseases.

To fill evidence gaps, we designed a large longitudinal cohort of neonates at moderate altitudes to investigate trajectories and to derive the lower limit of SpO_2_, taking advantage of the repeated measurements at seven time points within the first 72 hours.

## Methods

### Participants

We carried out this study at The People's Hospital of Luchun County at an altitude of 1650 m above sea level between January 1, 2020 and July 1, 2020 (ClinicalTrials.gov identifier: NCT04238104). Luchun County is located in southern Yunnan Province and has been identified as one of the most deprived counties in China. Approximately 80% of all newborns in the county were delivered in The People's Hospital, where the newborn unit in the area was located (average of 2300 deliveries annually). All consecutive newborns who did not require supplemental oxygen were enrolled and measured for pulse oximetry measurements initiated at 6 hours after birth, irrespective of gestational age and birth weight. Among them, those with hypoxemic diseases identified before discharge via clinical symptoms (cyanosis, lethargy, apnea, poor feeding, tachypnea, cough) combined with auxiliary examinations (X-ray for pneumonia, enhanced computed tomography for pulmonary arteriovenous malformation, laboratory tests for sepsis and polycythemia) were immediately transferred to the neonatal ward for necessary treatment. Accordingly, neonates without hypoxemic conditions comprised the asymptomatic cohort for the analysis of SpO_2_ trajectory patterns. In China, neonatal CHD screening based on pulse oximetry and clinical assessment has been incorporated into the universal newborn screening program since 2018. It was standard in Luchun County when our study commenced and the need for informed consent was waived. This study was approved by the Ethics Committee of Children's Hospital of Fudan University (institutional review board number: 2020132).

### Procedures

Pre- and post-ductal SpO_2_ values were measured repeatedly at seven time points at 6, 12, 18, 24, 36, 48, and 72 hours after birth, according to the length before hospital discharge. A Masimo Radical-7 pulse oximeter and LNCS Y1 reusable probe were placed on the right hand and in close succession on a single foot by trained obstetric nursing staff. Stringent quality control measures were included in the standard operating procedures for quality assurance of SpO_2_ data. First, whenever possible, we attempted to screen neonates in the supine position, sleeping or awake and quiet without concurrent feeding, fuss, or crying. Second, oximetry readings were recorded after having a stable and sharp pulsatile pulse waveform for one minute. Third, it is worth noting that any potentially abnormal measurement (i.e., either pre- or post-ductal readings < 95% or a > 3% difference between the two) was double checked on the spot by at least one trained staff member. This was done by reconfirming the proper placement of the probe and reading the records until the highest stable level lasted for at least two minutes. When discordance between the two records existed, the reconfirmed one was adopted.

All infants underwent echocardiography before discharge with blinded SpO_2_ measurements and those identified with CHDs received subsequent echocardiography during the routine 42-day childcare follow-up. Newborns who presented with any disease symptoms after discharge were referred for assessment to the hospital, as it is the only institution providing neonatal medical services in the country. As such, all neonatal health status information, including the subsequent diagnosis of hypoxic disease, was available in the hospital information system, enabling the ascertainment of all neonates' health conditions.

### Statistical analyses

We presented the characteristics of the study neonates as a whole and separately by hypoxemic conditions. We summarized continuous variables by the mean and standard deviation (SD) or median and interquartile range (IQR) as appropriate and categorical variables by frequency and proportion. We descriptively analyzed neonates with hypoxemic diseases. We used a linear mixed-effects model to examine the trajectories of SpO_2_ within the first 72 hours [17]. The model included fixed effects for measuring position (pre-ductal vs. post-ductal), dummy-coded time effects for each time point (6, 12, 18, 24, 36, 48, and 72 hours), infant sex (male vs. female), delivery mode (natural vs. cesarean), low birth weight (< 2500 vs. ≥ 2500 g), preterm status (< 37 vs. ≥ 37 gestational weeks), and random effects for a normally distributed intercept for each neonate and linear time. To account for possible dependence on repeated births from the same mother, we constructed models with the maternal identification number as a cluster variable. We used unstructured covariance determined by Akaike information criteria [18]. We presented SpO_2_ trajectories over time stratified by measuring position and examined whether the trajectories differed by testing the significance of the position-by-time interaction term using the Wald test.

We used the LMS values and penalized smooth percentiles for SpO_2_ over time separately for pre- and post-ductal positions (LMS Chart Maker Light V.2.3, Institute of Child Health, London, England). The 2.5th percentile of SpO_2_ distribution was considered the SpO_2_ cutoff for identifying hypoxemic diseases in this study. We additionally assessed the 97.5th percentile of the distribution of the difference between pre- and post-ductal SpO_2_ as the cutoff value to inform potential CCHD screening strategies. All analyses were performed by STATA 16.0 (Stata Corp LP, College Station, TX, USA). A two-tailed *P* value of < 0.05 was regarded as statistically significant.

## Results

**Fig. 1:**
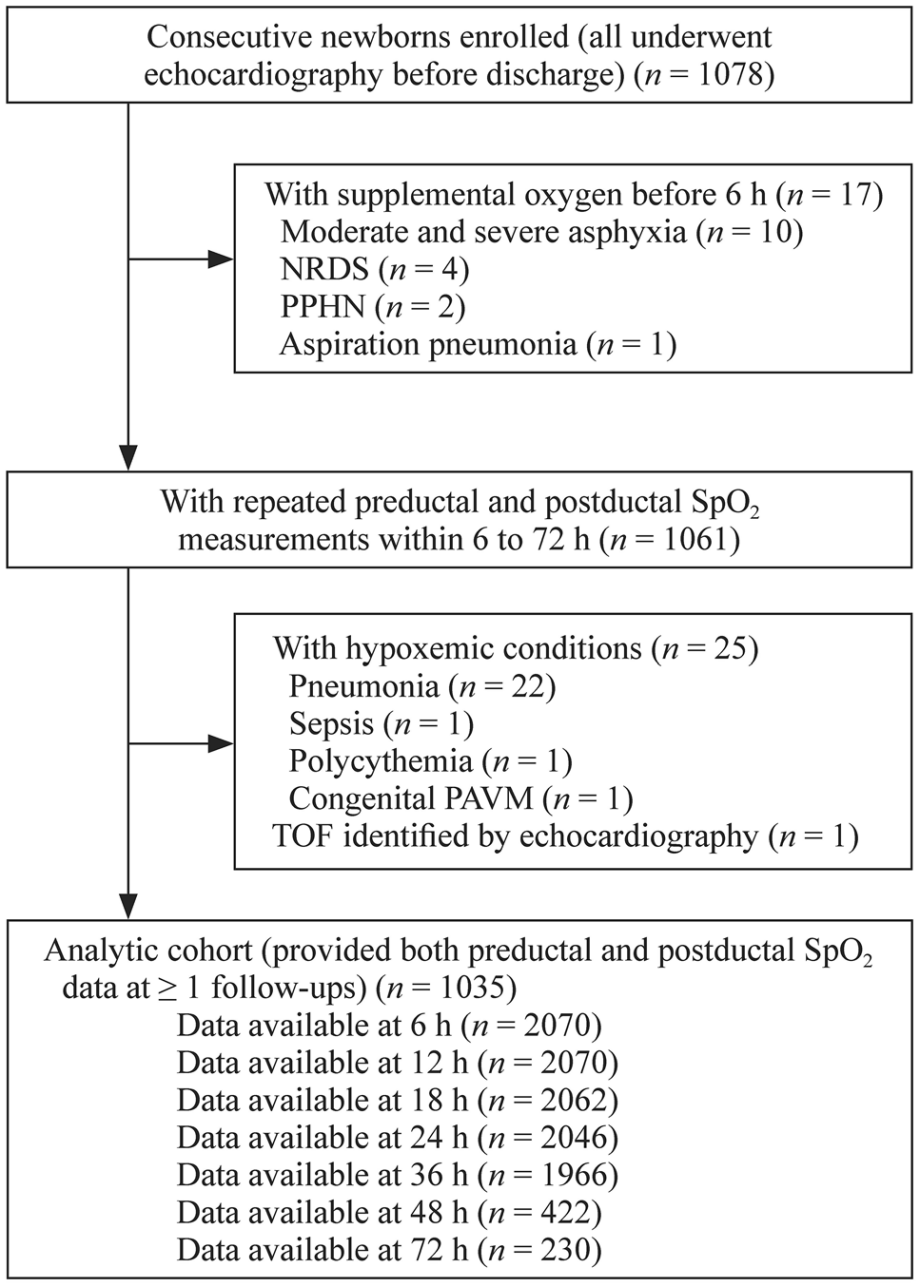
Recruitment and follow-up SpO_2_ measurements for all neonates. *SpO*_*2*_ pulse oxygen saturation, *NRDS* neonatal respiratory distress syndrome, *PPHN* persistent pulmonary hypertension of the newborn, *TOF* tetralogy of Fallot, *PAVM* pulmonary arteriovenous malformation

Between January and July 2020, 1078 consecutive newborns were delivered to the study hospital, and all underwent echocardiography before discharge. Among them, 1061 neonates without supplemental oxygen requirements were included and repeatedly measured for SpO_2_ within 72 hours (Fig. 1). During this period, 25 neonates with non-cardiac hypoxemic conditions were identified, including one case of sepsis, one case of polycythemia, one case of pulmonary arteriovenous malformation, and 22 cases of pneumonia (Table 1). The mean age at symptom onset was 22.8 hours, and that at the first detection of abnormal SpO_2_ was 18.4 hours. All cases were asymptomatic when abnormal SpO_2_ was first detected, with 84% (21/25) aged ≤ 24 hours. One neonate with tetralogy of Fallot was identified with echocardiography (Table 2). This resulted in 1035 asymptomatic neonates for the analysis of assessing the trajectories of SpO_2_ within 72 hours after birth (Fig. 1).

**Table 1:** Neonates presenting with non-cardiac hypoxemic diseases within 6 to 72 hours after birth

No.	Diagnosis^a^	Symptoms	Time of symptom onset (h)	Abnormal SpO_2_ detected (h)	Pre-/post-ductal SpO_2_ (%)
1	Congenital PAVM	Cyanosis	13	6	89/88
2	Pneumonia	Lethargy	18	6	91/92
3	Pneumonia	Poor feeding	10	6	91/92
4	Pneumonia	Abdominal distension	10	6	86/84
5	Pneumonia	Tachypnea	14	12	90/88
6	Pneumonia	Apnea	28	12	91/89
7	Pneumonia	Lethargy	17	12	90/90
8	Pneumonia	Tachypnea	14	12	89/89
9	Pneumonia	Poor feeding	16	12	90/89
10	Pneumonia	Poor feeding	15	12	92/90
11	Pneumonia	Cough	20	18	90/92
12	Pneumonia	Lethargy	22	18	90/99
13	Pneumonia	Cough	21	18	90/90
14	Pneumonia	Lethargy	27	18	92/90
15	Pneumonia	Lethargy	24	18	95/91
16	Pneumonia	Apnea	34	18	90/94
17	Pneumonia	Cough	20	18	92/89
18	Pneumonia	Apnea	23	18	92/90
19	Pneumonia	Tachypnea	26	24	90/88
20	Pneumonia	Apnea	30	24	92/91
21	Sepsis	Poor feeding	25	24	89/88
22	Pneumonia	Tachypnea	40	36	89/92
23	Pneumonia	Poor feeding	38	36	91/91
24	Pneumonia	Abdominal distension	27	36	90/90
25	Polycythemia	Lethargy	38	36	84/90

*SpO*_*2*_ pulse oxygen saturation, *PAVM* pulmonary arteriovenous malformation. ^a^Identified before discharge via clinical symptoms (cyanosis, lethargy, apnea, poor feeding, tachypnea, cough) combined with auxiliary examinations (X-ray for pneumonia, enhanced computed tomography for PAVM, laboratory tests for sepsis and polycythemia)

**Table 2:** Congenital heart disease identified by echocardiography

Subtype	Before discharge^a^	At 42 d follow-up^b^
Ventricle septal defect	11	9
Atrial septal defect	15	7
Patent ductus arteriosus	747	13
Mild aortic coarctation	2	1
Tetralogy of Fallot^c^	1	1

*CHD* congenital heart disease. ^a^Echocardiography was performed on all the 1078 neonates recruited at a mean of 17 hours after birth, and those with CHDs were listed; ^b^echocardiography was performed on those with CHDs identified before discharge to confirm their status; ^c^this neonate had normal oxygen saturation values before discharge

The characteristics of the study neonates are shown in Table 3. Overall, 52.7% were boys with a mean (SD) gestational age of 39.3 (1.4) weeks. Approximately 5.5% of the neonates were preterm, and 5% were of low birth weight. The length of hospitalization was 36 hours (IQR = 28.2–43.6 hours), with echocardiography performed at a median of 17.5 hours (IQR = 10.0–23.1 hours). Neonates with hypoxemic diseases were generally similar to their asymptomatic counterparts, except with a higher proportion of boys (80.8% vs. 52.0%, *P* = 0.004) and a higher birthweight (3442 vs. 3185 g, *P* = 0.004).

**Table 3:** Characteristics of the study neonates

Characteristics	Overall (*n* = 1061)	Neonates with hypoxemic diseases (*n* = 26)^b^	Asymptomatic neonates (*n* = 1035)	*P*^c^
Sex, *n* (%)				
Boys	559 (52.7)	21 (80.8)	538 (52.0)	0.004
Girls	502 (47.3)	5 (19.2)	497 (48.0)	
Gestational age (wk), mean (SD)	39.3 (1.4)	39.9 (1.1)	39.3 (1.5)	0.031
< 37	7 (0.7)	0 (0.0)	7 (0.7)	0.690
37–42	51 (4.8)	0 (0.0)	51 (4.9)	
> 42	1003 (94.5)	26 (100.0)	977 (94.4)	
Birth weight (g), mean (SD)	3192 (444)	3442(433)	3186 (442)	0.004
< 2500	53 (5.0)	0 (0.0)	53 (5.1)	0.057
2500 to < 4000	973 (91.7)	23 (88.5)	950 (91.8)	
≥ 4000	35 (3.3)	3 (11.5)	32 (3.1)	
Apgar 5 min	9.0 (9.0–9.0)	9.0 (9.0–9.0)	9.0 (9.0–9.0)	0.370
Heart rate (bpm), mean (SD)	129 (11)	132 (8)	129 (11)	0.130
Peripheral perfusion index (%), median (IQR)	1.9 (1.3–2.5)	2.0 (1.3–2.6)	1.9 (1.3–2.5)	0.580
Delivery mode, *n* (%)				
Natural	923 (87.0)	23 (88.5)	900 (87.0)	0.990
Cesarean	138 (13.0)	3 (11.5)	135 (13.0)	
Twin siblings, *n* (%)^d^	21 (2.0)	0 (0.0)	21 (2.0)	0.990
Time of echocardiography (h), median (IQR)	17.5 (10.0–23.1)	15.9 (8.6–23.2)	17.5 (10.0–23.1)	0.510
Length of hospitalization (h), median (IQR)	36.0 (28.2–43.6)	33.3 (25.3–61.1)	36.0 (28.2–43.6)	0.420

*SD* standard deviation, *IQR* interquartile range. ^a^Included ten organ anomalies: five extracardiac anomalies, two polydactylisms, one ankylotia, one anal atresia, and one absence of the left palm; ^b^twenty-five neonates with non-cardiac hypoxemic diseases presented within 6–72 hours after birth, and one with tetralogy of Fallot identified by echocardiography before discharge; ^c^*P* values for group comparisons were derived using the Student’s *t* test and Kruskal–Wallis test for continuous variables with and without normal distributions, and Pearson's Chi-square test and Fisher's exact test for categorical variables, as appropriate; ^d^ten pairs of twins were included in our study; one more neonate had a twin sibling, but his twin sibling was excluded from the study because of the presence of pneumonia

**Fig. 2:**
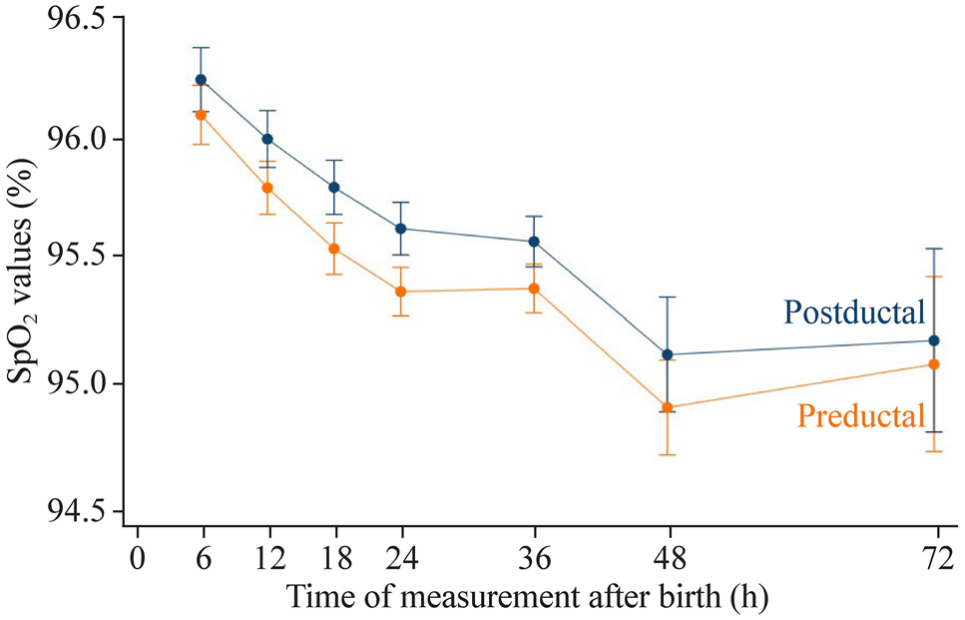
Estimated trajectories by measuring position for pulse oxygen saturation (SpO_2_) values within 6 to 72 hours after birth in 1035 asymptomatic neonates with repeated measurements. The estimate was derived from a linear mixed model that included fixed effects for measuring position, measuring time, the position-by-time interaction term, random effects for neonates and linear time, and the clustering effect of neonates delivered by the same mother. Error bars indicate 95% confidence intervals. The number of neonates at each time point was 1035 at 6 hours, 1035 at 12 hours, 1031 at 18 hours, 1023 at 24 hours, 983 at 36 hours, 211 at 48 hours, and 115 at 72 hours

Among the asymptomatic neonates, 10,866 records of pre- and post-ductal SpO_2_ were available, with 2070, 2070, 2062, 2046, 1966, 422, and 230 observations at 6, 12, 18, 24, 36, 48, and 72 hours after birth (Fig. 1). The mean (SD) pre- and post-ductal SpO_2_ values were 95.6% (1.7%) and 95.8% (1.8%), respectively. Estimated trajectories by measuring position for SpO_2_ across the seven time points are shown in Fig. 2. Pre-ductal SpO_2_ values decreased steadily from 6 hours after birth, reached a nadir at 48 hours, and tended to level off after that. Post-ductal SpO_2_ values followed an identical pattern but were generally higher than pre-ductal values [mean difference = 0.20%, 95% confidence interval (CI) = 0.15–0.26, *P* < 0.001; *P* for interaction for time trend = 0.75]. We observed a positive association between cesarean mode and SpO_2_ values (vs. natural delivery: mean difference = 0.52%; 95% CI = 0.32–0.72) but not for sex, low birth weight, and preterm births (Table 4).

**Table 4:** Associations between characteristics and SpO_2_ values in asymptomatic neonates (*n* = 1035)

Characteristics	Mean difference (95% CI)^a^	*P*
Measuring position (post-ductal vs. pre-ductal)	0.20 (0.16–0.25)	< 0.001
Measuring time (h)		
6	1.00	
12	− 0.27 (− 0.39 to − 0.15)	< 0.001
18	− 0.49 (− 0.61 to − 0.37)	< 0.001
24	− 0.66 (− 0.79 to − 0.53)	< 0.001
36	− 0.68 (− 0.81 to − 0.55)	< 0.001
48	− 1.21 (− 1.44 to -0.99)	< 0.001
72	− 1.09 (− 1.44 to -0.74)	< 0.001
Sex (girls)	0.09 (− 0.04 to 0.22)	0.177
Cesarean	0.52 (0.32–0.72)	< 0.001
Low birth weight (< 2500 g)	− 0.26 (− 0.60 to 0.09)	0.150
Gestational age (wk)		
< 37	− 0.12 (− 0.43 to 0.18)	0.426
37–42	1.00	
> 42	− 0.53 (− 1.41 to 0.36)	0.243

*SpO*_*2*_ pulse oxygen saturation, *CI* confidence interval. ^a^Mean difference and 95% CIs were estimated from a linear mixed model adjusting for all the characteristics listed. The position-by-time interaction term was not included in the final model because the term was not significant (*P* for interaction over time = 0.75)

**Fig. 3:**
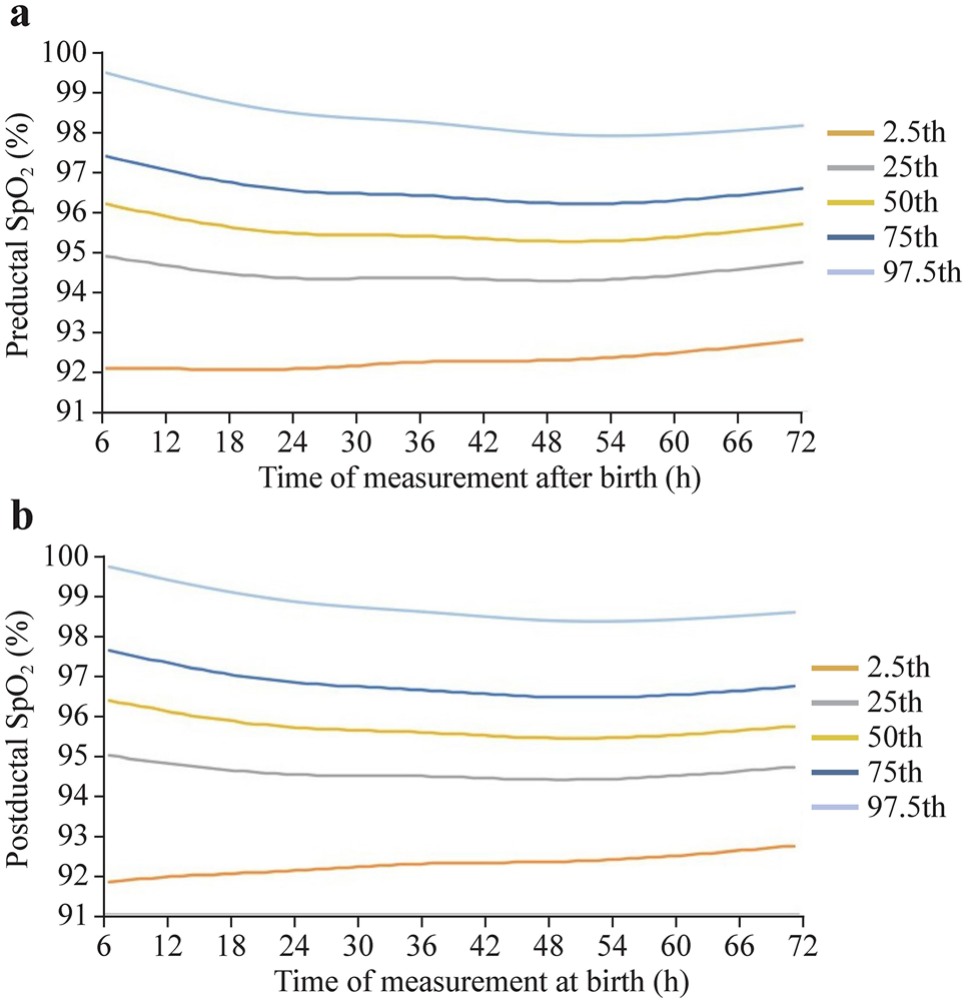
LMS curves for (**a**) pre-ductal SpO_2_ and (**b**) post-ductal SpO_2_ values. *SpO*_*2*_ pulse oxygen saturation

Pre- and post-ductal smoothed SpO_2_ percentiles throughout 6–72 hours after birth in asymptomatic neonates are shown in Fig. 3. The 2.5th percentile of the SpO_2_ distribution was 92% for both positions and turned out to be practically the same for all seven time points measured within the first 2 days. The 97.5th percentile of the pre- and post-ductal difference distribution was 3%.

## Discussion

In this large longitudinal cohort study, we demonstrated a pattern of decreasing SpO_2_ from 6 to 48 hours, followed by a flattening trend after birth in neonates at moderate altitudes. We subsequently derived the lower limit of 92% SpO_2_, which was time independent and could be used to inform suspected hypoxemic conditions. We found that most non-cardiac hypoxemic diseases presented with low SpO_2_ within 24 hours of life, with the timing noticed earlier than the onset of symptoms. The findings underscore the importance of pulse oximetry in timely identification of hypoxemic diseases at moderate altitudes and argue favorably for earlier CCHD screening.

The decreased trend in SpO_2_ values within 6 to 48 hours in our study population contradicts the general concepts of a presumably lower SpO_2_ within the first 24 hours at sea level [19]. Nevertheless, it corresponds to what has been reported by Niermeyer and colleagues at altitudes above 3100 m [6–8]. Similar findings were seen from a large cross-sectional study at 1800 m that the highest median SpO_2_ was noticed at 6 hours of life and is supposed to be stable and within the normal range [9]. Despite a prolonged transitional period after birth due to lower atmospheric pressure, saturation could still be attainable at moderate altitudes within a few hours after birth [20]. The potential physiological mechanism underlying the declining trend could be a more prominent periodic breathing at higher altitudes that produces greater desaturation cycles [8, 21, 22]. Newborn babies have a tremendous drive to breathe as a survival mechanism; that drive is mediated by an outpouring of stress hormones and thyroid hormones during birth, as well as a number of environmental factors. As that drive to breathe lessens over the first several days, saturations fall slightly at high altitudes. The same process likely occurs at sea level, but saturations are on the flat part of the oxyhemoglobin desaturation curve, and the change in saturation is not apparent.

The appropriate timing to perform pulse oximetry is a focus of persistent debate in CCHD screening [1, 10]. When screening earlier has been associated with a higher false positive rate compared with later screening at sea levels, our data suggest that this may not be the case at moderate altitude considering the relatively higher SpO_2_ within the first 24 hours. In some countries, such as the UK, mothers and infants are commonly discharged from the hospital within 24 hours after birth. In these circumstances, later screening is not practical, and most neonatal units in the UK screen within 24 hours of birth [23]. An additional consideration is a need to identify non-cardiac conditions that are also usually present in the first 24 hours, such as sepsis, pneumonia, and persistent pulmonary hypertension [12]. This figure was more than four-fifths of the non-cardiac diseases identified in our study population. Such considerations would have broad implications for less developed regions, where the timely recognition of hypoxemic diseases could have significant ramifications for improving overall public health [24, 25].

It is important to note that screening earlier than 24 hours at higher altitudes may sample when mean saturations are higher, but the saturation value will still be lower than at sea level. This again highlights the importance of adopting an altitude-suited SpO_2_ threshold value for screening [26]. Relative to the mean SpO_2_ of 98.5% at sea level, SpO_2_ around 24 hours of age at 1600–1900 m was lower and ranged from 93.0% to 97.2% [9, 16, 27–32]. Our mean SpO_2_ values of 95%–96% agreed with most of the prior studies [9, 27, 28, 30, 31], and we took advantage of repeated measurements with a motion-resistant pulse oximeter and a double-check procedure, thus largely minimizing the detection errors. The 95% SpO_2_ threshold value has been successfully applied in identifying hypoxemic conditions at sea level [1]. However, screening has not been widespread for regions at higher altitudes, plausibly due to the lack of well-recognized screening threshold values. We are aware of only two studies that have applied lower SpO_2_ threshold values at higher altitudes: 93% for screening 656 infants at 1818 m and 90% for 963 newborns at 2820 m within 24–96 hours after birth [11, 15]. However, these values were selected experientially, and their suitability for the data is unclear. Guo and colleagues proposed a lower limit (2.5th percentile) of 92% SpO_2_ at 24 hours after birth based on a large sample of 21,789 neonates at moderate altitudes (1500–2500 m), but it remains unresolved as to whether the lower limit varies within 24 hours [31]. Our current work takes the field of research one step closer. With repeatedly measured SpO_2_ across sufficient time points, we demonstrated that a lower limit of 92% SpO_2_ derived from our study population was time independent. Furthermore, a > 3% difference between pre- and post-ductal SpO_2_ derived from our study population was practically the same as that currently proposed at sea level.

To our knowledge, this was the largest longitudinal study of SpO_2_ trajectories within the first few days at moderate altitude. The rigorous study design, stringent measurements, and accurate diagnosis of hypoxemic conditions increased our capacity to characterize the trajectories unequivocally. Our findings will contribute to international efforts to implement pulse oximetry screening for CCHD at altitudes. Large CCHD screening studies at moderate altitudes are warranted to validate whether earlier screening would not produce higher false positives and could timely identify more non-cardiac hypoxemic diseases. Our study has limitations. The leveled-off trend observed at 48 and 72 hours needs to be interpreted with caution because of the relatively small number of neonates available, mainly due to the local postpartum care for mothers and infants with delivery modes. In Luchun County, naturally delivered mothers and infants are commonly discharged from the hospital within 24–36 hours, and those with cesarean delivery are discharged at 48 hours or later. Nevertheless, such a trend is supported by Niermeyer and colleagues' work that may continue at least one week after birth [6–8]. Additionally, despite being minor, the issue of ethnic disparities cannot be neglected [6], and whether our findings apply to other ethnicities or populations needs further investigation.

In conclusion, we provided a complete picture of the trajectories of SpO_2_ within the first 72 hours, featuring a decline within 48 hours. Combined with the observation that most non-cardiac hypoxemic diseases occurred within 24 hours, our findings suggest that an earlier screening within 24 hours with an altitude-suited SpO_2_ cutoff value could favorably achieve a benefit–risk balance in identifying hypoxemic diseases in this population.

## Data Availability

The datasets generated and analyzed during the current study are available from the corresponding author upon reasonable request.
